# Supplementary material for: KLB, encoding β‐Klotho, is mutated in patients with congenital hypogonadotropic hypogonadism
Source: EMBO Mol Med. 2017 Jul 28;9(10):1379–97. doi: 10.15252/emmm.201607376 (PMC5623842; doi:10.15252/emmm.201607376)
Supplement: Supplementary file 1 — Appendix [file EMMM-9-1379-s001.pdf]

Appendix

Table of content:

1. Appendix Case Reports
2. Appendix Table S1. FGFR1 mutations identified in patients with congenital hypogonadotropic hypogonadism (n=334)
3. Appendix Table S2. Primers for gene expression study
4. Appendix Table S3. Detailed statistical results
5. Appendix Reference

Appendix Supplementary Case Reports

Proband 1: The Caucasian male proband had a history of unilateral cryptorchidism (surgically corrected at 18 months) and came to medical attention in his mid-20s for complete absence of puberty (testicular volume 1-2 ml bilaterally). His serum testosterone level was low (<1 nmol/l) and gonadotropins were undetectable (LH and FSH both < 1IU/L). Pituitary MRI was normal and other pituitary functions were within normal limits, consistent with isolated GnRH deficiency. Based on his self-reported anosmia he was diagnosed with Kallmann Syndrome (KS). He was obese (height 174cm, weight 122kg, BMI 40.3kg/m^2) with insulin resistance (fasting glucose 5.3mmol/l, fasting insulin 124pmol/l, HOMA-IR 4.85). The lipid profile was within normal limits. The proband's brother was also obese and diagnosed with KS. A heterozygous *KLB* mutation (p.R309W) was identified in both the proband and his KS brother (Figure 2B, Family 1). No other mutations in CHH genes were identified.

Proband 2: The Caucasian male proband came to medical attention at age 16 for delayed puberty and was treated with testosterone injections which induced virilization. At age 28, he discontinued treatment for re-evaluation. He was frankly hypogonadal (T 0.4 nmol/L) in the setting of undetectable gonadotropins (LH & FSH <1.6 IU/l). His testicular volume was 9 ml bilaterally. Formal smell testing confirmed anosmia (UPSIT 25/40, < 5%ile) and a MRI revealed bilateral hypoplastic olfactory bulbs and tracts. Based on this presentation he was diagnosed with Kallmann syndrome and initiated pulsatile GnRH therapy. On treatment he maintained normal testosterone levels, developed fertility (maximal sperm count 80 X 10^6/mL) and fathered two children. He was normal weight (weight 83kg, height 178cm, BMI 24.7 kg/m^2), with high fasting glucose level at 5.6 mmol/L, yet normal fasting insulin level at 4.6 IU/L (HOMA-IR 1.14). Notably, his father has a diminished sense of smell. The proband and an unaffected brother harbor a heterozygous *KLB* mutation (p.R309Q), inherited from his father. In addition, the proband and his father harbor a heterozygous loss-of-function mutation in *FGF8* (p.P26L) (Falardeau et al, 2008).

Proband 3: The Caucasian male proband was born with unilateral cryptorchidism and underwent orchidopexy at age 12. He presented to medical attention at age 31 for his lack of secondary sexual characteristics. Serum testosterone was hypogonadal (1.5 nmol/L) with low gonadotropins (LH and FSH both 2 IU/l) in the setting of otherwise normal pituitary function. Based on these results and his absent sense of smell he was diagnosed with Kallmann syndrome. He was overweight (height 178cm, weight

88kg, BMI 27.9kg/m^2) yet no additional metabolic investigations were performed at that time. In terms of his family history, the proband's father was anosmic yet evidently had a normal puberty and fertility. The proband harbors a heterozygous mutation in *KLB* (p.R424C) without other mutations in known CHH genes.

Proband 4: The proband of South Asian descent (Indian subcontinent) was noted to have micropenis (without cryptorchidism) at birth and received testosterone treatment during infancy. At age 19 he presented for evaluation of absent pubertal development (testicular volume 2ml bilaterally). His serum testosterone and gonadotropins were undetectable and otherwise normal pituitary function and pituitary MRI. He presented mild scoliosis, without other CHH associated phenotypes. He reported a normal sense of smell which was confirmed by formal testing (UPSIT 39/40, >50%ile, normal). He is normal weight (height 178cm, weight 70kg, BMI 22.2 kg/m^2) with dyslipidemia (elevated triglycerides 270 mg/dl, total cholesterol 217 mg/dl and LDL 164 mg/dl), and his fasting glucose was high (5.7 mmol/l). The family history is positive for delayed puberty in the father. Both the proband and his father harbor a heterozygous mutation in *KLB* (p.A574T). The proband also harbor a heterozygous mutation in *PROKR2* (p.S188L), inherited from his mother (Cole et al, 2008).

Proband 5: The Caucasian female proband came to medical attention for absent pubertal development and primary amenorrhea at age of 18. Hormonal testing revealed low serum estradiol in the setting of low LH and FSH levels and otherwise normal pituitary function. She reported normal sense of smell and exhibited no other CHH-associated phenotypes. She had normal weight (height 170cm, weight 70kg, BMI 22.6kg/m^2), but was not evaluated for other metabolic profile. Notably, her brother has normosmic CHH and they both harbor a heterozygous deletion in *KLB* (p.F777delF) inherited from the father as well as a heterozygous mutation in *GNRHR* (p.Q106R) inherited from the mother (Figure 2, Family 5). GNRHR p.Q106R was reported to be partial loss-of-function in *in vitro* assays and reported in several CHH patients (de Roux et al, 1997).

Proband 6: The Caucasian female proband came to medical attention at age 16 for evaluation of primary amenorrhea. On exam she was noted to have crowded teeth, some signs of adrenarche (axillary and Tanner III pubic hair) yet lacked signs of estrogenization (Tanner I breasts). Serum hormonal measurement revealed low estradiol (11 pg/ml) with low LH (1.0 IU/L) and FSH (2.0 IU/L) and otherwise normal pituitary function. MRI revealed a small yet otherwise unremarkable pituitary. While she reported a normal sense of smell, formal olfactory testing indicated hyposmia (UPSIT 33/40, 7%ile) consistent with a diagnosis of Kallmann syndrome. She was underweight (height 151.6cm, weight 38.2kg, BMI 16.6kg/m^2), with normal fasting glucose (4.4mmol/l), but no further metabolic investigations were performed. Her family history was remarkable for delayed puberty in the proband's brother and mother. The proband, her

mother and brother all harbor a heterozygous *KLB* mutation (p.F777delF), with no other mutations in known CHH genes (Figure 2, Family 6).

Proband 7: The Caucasian male proband was born with bilateral cryptorchidism and micropenis and was followed by the pediatric endocrine service for suspicion of CHH. When he was identified as being anosmic at age 7, the presumptive diagnosis of Kallmann syndrome was made. At age 13.5 when he failed to exhibit any signs of spontaneous puberty he was started on testosterone treatment to induce development of secondary sexual characteristics. After attaining his final height (179 cm) treatment was stopped and hormonal evaluation confirmed the diagnosis. At that time he was obese (BMI 42.5 kg/m^2), with insulin resistance (fasting glucose 5.2 mmol/l, insulin 109 pmol/l, HOMA-IR 3.6) and dyslipidemia (triglyceride 1.8 mmol/l, cholesterol 5.4 mmol/l). Notably, the proband's two paternal great uncles and a paternal great great uncle also had Kallmann syndrome. The proband harbors a heterozygous deletion in *KLB* (*p.F777delF*) inherited from his unaffected mother who had normal puberty and fertility. No other mutations in known CHH genes were identified in the proband.

Proband 8: The Caucasian male proband was born with colorblindness and bilateral polydactyly (surgically corrected during childhood). He came to medical attention age 19 for arrested pubertal development and no ejaculation. At that time, his serum testosterone was low (3.6 nmol/L) with abnormally normal LH (2.3 IU/L) and FSH (4 IU/L). The physical examination was remarkable for partial pubertal development (testicular volume 13mL bilaterally). He reported absent sense of smell, confirmed by formal olfactory testing (B-SIT 2/12, <5%ile) and cranial MRI revealed olfactory bulb agenesis, consistent with the diagnosis of Kallmann Syndrome. He was normal weight (BMI 20.9 Kg/m^2) with normal fasting glucose (4.6 mmol/L) yet elevated serum triglyceride levels (210 mg/dL). He initiated testosterone replacement with good effect then subsequently underwent a treatment washout in advance of a neuroendocrine evaluation. Off treatment it was discovered that he had recovered function of his hypothalamic-pituitary-gonadal (HPG) axis as evidenced by pulsatile LH secretion (6 pulses in 12 hrs, mean LH 6.4IU/L, FSH 7.1 IU/L), normal serum testosterone level (13.9 nmol/L) and active spermatogenesis (65 X 10^6/mL) (Raivio et al, 2007). Family history was unremarkable. The proband was found to harbor a heterozygous mutation in *KLB* (p.F777delF) inherited from his unaffected mother. No other mutations in known CHH genes were identified in the proband.

Proband 9: The male Caucasian proband was born with retractile testes and micropenis. The neonatal presentation raised the suspicion of CHH and when he failed to initiate spontaneous puberty he was diagnosed with Kallmann syndrome based on his hormonal profile and anosmia (B-SIT 6/12, <5%ile). Ultrasound revealed a renal diverticulum and a double ureter. At 16 years old, he was overweight (BMI 24.6kg/m^2, >95%ile for age). He had elevated fasting glucose (6.6 mmol/L) and triglycerides (193 mg/dL). There was no family history of delayed puberty, infertility, anosmia or urogenital problems. He harbors a

heterozygous deletion in *KLB* (p.F777delF) as well as a heterozygous mutation in *FGFR1* (p.R78C) (Pitteloud et al, 2006).

Proband 10: The Caucasian male proband was found to have inguinal testes identified during a sports physical at age 12. He lacked pubertal development and his bone age was delayed by 2 years. Hormonal evaluation indicated frank hypogonadism (testosterone 0.3 nmol/L) and undetectable gonadotropins. He underwent a bilateral orchidopexy at age 13 and initiated testosterone therapy leading to develop secondary sexual characteristics. At age 25, he stopped testosterone treatment for re-evaluation. His testicular volume was prepubertal (<1 mL bilaterally), he exhibited bilateral synkinesia, and he was anosmic (UPSIT10/40, <5%ile). Serum hormone measurements confirmed hypogonadotropic hypogonadism (T 0.4 nmol/L, LH 1.7 IU/L, FSH 1.6 IU/L) and he was diagnosed with Kallmann syndrome. At that time he was overweight (BMI at 26.3 kg/m^2), yet no further metabolic evaluation was performed. The family history was noncontributory. The proband was found to harbor a heterozygous deletion in *KLB* (p.F777delF) without mutations in any other CHH loci.

Proband 11: The Caucasian male proband was born with distal hypospadias and unilateral cryptorchidism (surgically corrected at 23 months). Additionally,he exhibited cleft palate, bifid uvula, hypertelorism and strabismus. His development was delayed with mental retardation and he presented at age 16 for failure to initiate puberty. At that time he had prepubertal testes (1ml bilaterally) and hormone assessment revealed hypogonadotropic hypogonadism (T < 1 nmol/L, LH 0.2 UI/L and FSH 0.5 UI/L). He self-reported anosmia and MRI revealed agenesis of the corpus callosum, cerebellar vermis (partial) and absent olfactory bulbs - consistent with Kallmann Syndrome. He had normal weight (BMI 19.5kg/m^2) with a normal lipid profile yet mild insulin resistance (fasting glucose 5.1 mmol/L, insulin 15.8 mU/L, HOMA-IR 3.6). There is no family history of pubertal delay, infertility or anosmia. He harbors heterozygous mutations in *KLB* (p.F777delF) and in *PROKR2* (p.L173R).

Proband 12: The Caucasian male proband presented at age 56 with a chief complaint of sexual dysfunction. He reported having congenital anosmia, had a history of delayed puberty, exhibited marked segmental disproportion (eunuchoidal) and had severe osteoporosis (lumbar T-sore -3.7, hip T score -2.9). Hormonal evaluation revealed hypogonadotropic hypogonadism (T 3.6 nmol/L, LH 1.9 IU/L, FSH 3.2 IU/L) and formal testing confirmed anosmia (UPSIT 7/40, <5%ile) and MRI indicated absent olfactory sulci. Notably, he stated that he had never received hormonal treatment yet he had fathered two children in his late 20s. Thus the history, clinical presentation and hormonal profile were consistent with the 'fertile eunuch' variant of Kallmann Syndrome (Santhakumar et al, 2014). There was no family history of pubertal delay, infertility or anosmia. He has normal weight (BMI 21.7 kg/m^2), normal body composition (total body fat 23%) with normal lipid profile (total cholesterol, HDL, triglycerides) as well as fasting glucose (4.8

mmol/L) and insulin (2.9 uIU/ml, HOMA-IR 0.62). He harbors a heterozygous mutation in *KLB* (p.K815E) with no other mutations in known CHH genes.

Proband 13: The Caucasian, Jewish male proband was born with congenital anosmia and micropenis without cryptorchidism. He was diagnosed with Kallmann Syndrome and started testosterone at 13 years old to induce growth and development of secondary sexual characteristics. At age of 19, he underwent a treatment washout prior to re-evalution. At that time his testicular volume was 4-5 mL bilaterally and he remained hypogonadal (T < 0.1 nmol/l) with low gonadotropins (LH and FSH 0.5 UI/L) and otherwise normal pituitary function - consistent with Kallmann syndrome. Physical examination was notable for a high arched palate and flat feet. The anosmia was confirmed by formal testing (B-SIT 4/12, <5%ile) and dysplasia of olfactory sulcus on MRI. Densitometry revealed osteopenia (lumbar T score -2.4, hip -1.8). At age of 19, he was normal weight (height 173cm, weight 59kg, BMI at 20 kg/m^2) and did not have any further metabolic testing. The family history is notable for isolated anosmia in the father. Additionally, the proband's sister exhibits anosmia combined with sensorineural hearing loss. She had delayed puberty (partial breast development and primary amenorrhea), but the final clinical diagnosis was not available. He harbors a heterozygous *KLB* mutation (p.L1011P) inherited from his mother.

Appendix Supplementary Tables

Appendix Table S1. *FGFR1* mutations identified in patients with congenital hypogonadotropic hypogonadism (n=334)

nucleotide change	protein change	type of mutation	Domain	Patient Dx	MAF (%) ExAC
c.232C>T	p.R78C	missense	IgD1	2 KS	0
c.296A>G	p.Y99C	missense	IgD1	1 KS, 1 nCHH	0
c.622-1G>T	-	splice_acceptor	IgD1	CHH	0
c.670G>C	p.D224H	missense	IgD2	KS	0
c.682T>G	p.Y228D	missense	IgD2	nCHH	0
c.709G>A	p.G237S	missense	IgD2	KS	0
c.710G>A	p.G237D	missense	IgD2	KS	0
c.749G>A	p.R250Q	missense	Between IgD2-D3	KS	0
c.761G>A	p.R254Q	missense	Between IgD2-D3	1 KS, 2 nCHH	0
c.790A>T	p.N264Y	missense	IgD3	KS	0
c.817G>A	p.V273M	missense	IgD3	KS	0
c.821A>G	p.E274G	missense	IgD3	KS	0
c.1016A>G	p.Y339C	missense	IgD3	KS	0
c.1025T>C	p.L342S	missense	IgD3	KS	0
c.1038_1039insTT	p.I347Lfs*11	frameshift	NMD	KS	0
c.1039_1040insT	p.G348Rfs*60	frameshift	IgD3	KS	0
c.1042G>A	p.G348R	missense	IgD3	3 KS	0
c.1070C>T	p.T357I	missense	IgD3	KS	0
c.1093_1094dupAG	p.P366Gfs*4	frameshift	NMD	KS	0
c.1286T>A	p.V429E	missense	FRS2 binding	KS	0
c.1306_1307dupTC	p.M437Pfs*2	frameshift	NMD	KS	0
c.1368G>T	p.M456I	missense	-	KS	0.006
c.1430+1delG	NA	splice_donor	TKD	KS	0
c.1447C>A	p.P483T	missense	TKD	KS	0
c.1549-2T>C	-	splice donor	TKD	nCHH	0
c.1755C>A	p.Y585*	nonsense	TKD	KS	0
c.1756_1763dupAACCCCAG	p.S588Rfs*47	frameshift	NMD	CHH	0.001
c.1864C>T	p.R622*	nonsense	NMD	1 KS, 1nCHH	0
c.1961dupA	p.Y654*	frameshift	NMD	KS	0
c.2011G>C	p.A671P	missense	TKD	nCHH	0
c.2038C>T	p.Q680*	nonsense	NMD	nCHH	0
c.2058delC	p.F686Lfs*28	frameshift	NMD	KS	0
c.2059G>A	p.G687R	missense	TKD	KS	0
c.2075A>G	p.E692G	missense	TKD	KS	0
c.2107G>C	p.G703R	missense	TKD	KS	0
c.2165C>A	p.P722H	missense	TKD	nCHH	0
c.2172C>G	p.N724K	missense	TKD	nCHH	0
c.2233C>T	p.P745S	missense	TKD	KS	0

Dx : diagnosis ; MAF : minor allele frequency ; CHH : congenital hypogonadotropic hypogonadism ; KS : Kallmann Syndrome ; the underlined mutation was found in homozygous status; IgD : immunoglobulin domain ; NMD : nonsense-mediated mRNA decay ; TKD : tyrosine kinase domain.

Appendix Table S2. Primers for gene expression study

Gene	Forward primer	Reverse primer
Hprt	5’-AGCAGTACAGCCCCAAAATGG-3’	5’- ATCCAACAAAGTCTGGCCTGTAT-3’
Rps29	5'-GCCAGGGTTCTCGCTCTTG-3'	5'-GGCACATGTTCAGCCCGTAT-3'
Gnrh1	5'-CGTTCACCCCTCAGGGATCT-3'	5'-TGCCTGGCTTCCTCTTCAAT-3'
Lepr	5'-TCAGAATTTTGGGTGGAAAA-3'	5'-GTCCAGGTGAGGAGCAAGAG-3'
Gpr54	5'-ACATACCAGCGGTCCACACT-3'	5'-GGTGCTGGGAGACTTCATGT-3'
Kiss1	5'-GCTGCTGCTTCTCCTCTGTGT-3'	5'-GGACTGCTGGCCTGTGGAT-3'
Avp	5’-CGCTCTCCGCTTGTTTCCT-3’	5’- CTCTTGGGCAGTTCTGGAAGTAG-3’
Pomc	5'-TGCTTCAGACCTCCATAGATGTGT-3'	5'-GGATGCAAGCCAGCAGGTT-3'
Npy	5'-TCTGCGACACTACATCAATCTCATC-3'	5'-GTGTCTCAGGGCTGGATCTCTT-3'
Klb	5'-AAGAGTCCACGCCAGACATGA-3'	5'-ACTCGGGCTTAAGAACAGACTCA-3'
Fgfr1	5’-CCAAACCGTAGGCCTGTAGCT-3’	5’- CATGCAGTTTCTTCTCCATTTTCTC-3’

Appendix Table S3. Detailed statistical results

Figure	Measurement	n	Comparison	P-val
1A	FGF8 signaling	n = 3<br>(for each conditions)	Maximal signaling: FGFR1 L342S vs WT<br>EC50: FGFR1 L342S vs WT	0.5440<br>5.57E-07
1B	FGF21 signaling	n = 3<br>(for each conditions)	Maximal signaling: FGFR1 L342S vs WT<br>EC50: FGFR1 L342S vs WT	0.0013<br>0.1611
2A	FGF21 signaling	n = 3<br>(for each conditions)	Maximal signaling: KLB R309W vs WT<br>EC50: KLB R309W vs WT<br>Maximal signaling: KLB L1011P vs WT<br>EC50: KLB L1011P vs WT<br>Maximal signaling: KLB F777delF vs WT:<br>EC50: KLB F777delF vs WT<br>Maximal signaling: KLB R424C vs WT<br>EC50: KLB R424C vs WT	0.2299<br>4.06E-05<br>7.68E-08<br>0.9986<br>2.86E-08<br>0.3816<br>1.14E-10<br>0.2650
2B	FGF21 signaling	n = 3<br>(for each conditions)	Maximal signaling: KLB R309Q vs WT<br>EC50: KLB R309Q vs WT<br>Maximal signaling: KLB A574T vs WT<br>EC50: KLB A574T vs WT<br>Maximal signaling: KLB K815E vs WT:<br>EC50: KLB K815E vs WT	0.0401<br>0.6279<br>0.0015<br>0.4654<br>0.0152<br>0.8972

2D	Cell surface expression	n = 3<br>(for each conditions)	R309W vs WT<br>R309Q vs WT<br>R424C vs WT<br>A574T vs WT<br>F777delF vs WT<br>K815E vs WT<br>L1011P vs WT	9.65E-05<br>8.06E-04<br>0.1245<br>0.0125<br>2.26E-05<br>4.76E-04<br>0.0029
2E	FGF21 signaling	n = 3<br>(for each conditions)	Maximal signaling:<br>FGFR1 WT + KLB F777delF vs. FGFR1 WT + KLB WT<br>FGFR1 R78C + KLB WT vs. FGFR1 WT + KLB WT<br>FGFR1 R78C + KLB F777delF vs. FGFR1 WT + KLB WT<br>FGFR1 R78C + KLB F777delF vs FGFR1 R78C + KLB WT<br>FGFR1 R78C + KLB F777delF vs. FGFR1 WT + KLB F777delF	 0.0308<br>5.68E-05<br>5.24E-10<br>0.0010<br>7.01E-09

2G	Rescue of cyst phenotype (*C. elegans*)	klo-2; klo1 KO, n = 174<br>pklo-1::huKLB WT, n = 146<br>pklo-2::huKLB WT, n = 98<br>R309W, n = 68<br>R309Q, n = 80<br>R424C, n = 102<br>A574T, n = 150<br>F777delF, n = 114<br>K815E, n = 155<br>L1011P, n = 146	klo-2; klo1 KO vs. pklo1::huKLB WT<br>pklo-2::huKLB WT vs. pklo1::huKLB WT<br>R309W vs. pklo1::huKLB WT<br>R309Q vs. pklo1::huKLB WT<br>R424C vs. pklo1::huKLB WT<br>A574T vs. pklo1::huKLB WT<br>F777delF vs. pklo1::huKLB WT<br>K815E vs. pklo1::huKLB WT<br>L1011P vs. pklo1::huKLB WT<br>pklo-1::huKLB WT vs. klo-1; klo-2 KO<br>pklo-2::huKLB WT vs. klo-1; klo-2 KO<br>R309W vs. klo-1; klo-2 KO<br>R309Q vs. klo-1; klo-2 KO<br>R424C vs. klo-1; klo-2 KO<br>A574T vs. klo-1; klo-2 KO<br>F777delF vs. klo-1; klo-2 KO<br>K815E vs. klo-1; klo-2 KO<br>L1011P vs. klo-1; klo-2 KO	1.19E-10<br>0.3854<br>1.66E-06<br>0.0152<br>1.99E-04<br>2.68E-04<br>1.34E-08<br>5.79E-05<br>8.11E-12<br>1.19E-10<br>2.01E-06<br>0.7556<br>0.0032<br>0.0484<br>0.0046<br>0.8961<br>0.0044<br>0.4754
4A	Vaginal opening	WT n = 11<br>KO n = 12	WT vs. KO	0.0029
4B	First estrus	WT n = 11<br>KO n = 7	WT vs. KO	0.0102

4C	Body weight curbe	WT n = 7<br>KO n = 7	P24: WT vs. KO<br>P25: WT vs. KO<br>P26: WT vs. KO<br>P27: WT vs. KO<br>P28: WT vs. KO<br>P29: WT vs. KO<br>P30: WT vs. KO<br>P31: WT vs. KO<br>P32: WT vs. KO<br>P33: WT vs. KO<br>P34: WT vs. KO<br>P35: WT vs. KO<br>P36: WT vs. KO	0.0038<br>0.0033<br>0.0015<br>0.0021<br>0.0027<br>0.0021<br>0.0018<br>0.0015<br>0.0014<br>0.0022<br>0.0021<br>0.0029<br>0.0029
4D	Body weight adult	WT n = 15<br>KO n = 10	WT vs. KO	3.40E-05
4E	Fat mass	WT n = 23<br>KO n = 20	WT vs. KO	0.5586
4H	Ovarian weight	WT n = 10<br>KO n = 7	WT vs. KO	0.047
4I	Corpora Lutea per ovary	WT n = 5<br>KO n = 5	WT vs. KO	0.041
4K	Time per estrous phase	WT n = 17<br>KO n = 13	Diestrus: WT vs. KO<br>Estrus: WT vs. KO	2.79E-08<br>2.79E-08
4L	Number of litter per female	WT n = 5<br>KO n = 6	WT vs. KO	0.0427
4L	Number of pups per litter	WT n = 5<br>KO n = 6	WT vs. KO	0.2450

4M	Preovulatory LH	WT Di n = 8<br>WT Pro n = 5<br>KO Di n = 5<br>Pro n = 5	Di:WT vs. Di:KO<br>Di:WT vs. Pro:WT<br>Di:WT vs. Pro:KO<br>Di:KO vs. Pro:WT<br>Di:KO vs. Pro:KO<br>Pro:WT vs. Pro:KO	0.8604<br>0.0051<br>0.5903<br>0.0144<br>0.7429<br>0.0291
4N	GnRH test	WT n = 7<br>KO n = 5	WT: basal vs. GnRH<br>KO: basal vs. GnRH	0.0024<br>0.0421
4O	Kiss test	WT n = 5<br>KO n = 5	WT: basal vs. Kiss<br>KO: basal vs. Kiss	0.0017<br>0.0147
5B	GnRH neuron distribution	WT n = 7<br>KO n = 4	-1080: WT vs. KO<br>-720: WT vs. KO<br>-360: WT vs. KO<br>ovlt: WT vs. KO<br>+360: WT vs. KO<br>+720: WT vs. KO<br>+1080: WT vs. KO<br>+1440: WT vs. KO	0.6888<br>0.7601<br>0.4565<br>0.9012<br>0.3845<br>0.3206<br>0.6575<br>0.3505
5C	GnRH neuron total number	WT n = 8<br>KO n = 4	WT vs. KO	0.6279

5D	Fgfr1 espression	Gn11, n = 4<br>GT1-7, n = 4<br>E13.5 nose, n = 4<br>E13.5 brain, n = 4<br>P0 POA, n = 4<br>P7 POA, n = 4<br>P21 POA, n = 4<br>Adult POA, n = 4	Gn11 vs. GT1-7<br>Gn11 vs. nose<br>Gn11 vs. brain<br>Gn11 vs. P0<br>Gn11 vs. P7<br>Gn11 vs. P21<br>Gn11 vs. Adult<br>GT1-7 vs. nose<br>GT1-7 vs. brain<br>GT1-7 vs. P0<br>GT1-7 vs. P7<br>GT1-7 vs. P21<br>GT1-7 vs. Adult<br>nose vs. brain<br>nose vs. P0<br>nose vs. P7<br>nose vs. P21<br>nose vs. Adult<br>brain vs. P0<br>brain vs. P7<br>brain vs. P21<br>brain vs. Adult<br>P0 vs. P7<br>P0 vs. P21<br>P0 vs. Adult<br>P7 vs. P21<br>P7 vs. Adult<br>P21 vs. Adult	5.30E-10<br>0.0014<br>7.97E-06<br>2E-14<br>4E-14<br>3.966E-11<br>2.71E-09<br>5.51E-07<br>1.37E-05<br>9.66E-07<br>3.42E-06<br>0.3791<br>0.1053<br>0.0665<br>2.4E-12<br>5.22E-12<br>3.28E-08<br>7.83E-06<br>8.66E-12<br>2.107E-11<br>5.81E-07<br>0.0004<br>0.5415<br>2.45E-06<br>8.28E-09<br>9.91E-06<br>2.68E-08<br>0.0108

5E	Klb expression	Gn11, n = 4<br>GT1-7, n = 4<br>E13.5 nose, n = 3<br>E13.5 brain, n = 3<br>P0 POA, n = 3<br>P7 POA, n = 6<br>P21 POA, n = 3<br>Adult POA, n = 4	Gn11 vs. GT1-7<br>Gn11 vs. nose<br>Gn11 vs. brain<br>Gn11 vs. P0<br>Gn11 vs. P7<br>Gn11 vs. P21<br>Gn11 vs. Adult<br>GT1-7 vs. nose<br>GT1-7 vs. brain<br>GT1-7 vs. P0<br>GT1-7 vs. P7<br>GT1-7 vs. P21<br>GT1-7 vs. Adult<br>nose vs. brain<br>nose vs. P0<br>nose vs. P7<br>nose vs. P21<br>nose vs. Adult<br>brain vs. P0<br>brain vs. P7<br>brain vs. P21<br>brain vs. Adult<br>P0 vs. P7<br>P0 vs. P21<br>P0 vs. Adult<br>P7 vs. P21<br>P7 vs. Adult<br>P21 vs. Adult	1.35E-05<br>0.9974<br>0.9978<br>9.36E-07<br>6.09E-08<br>1.86E-06<br>1.38E-09<br>3.65E-05<br>3.59E-05<br>0.1317<br>0.0727<br>0.2204<br>2.53E-04<br>0.9954<br>2.53E-06<br>2.69E-07<br>4.88E-06<br>5.54E-09<br>2.49E-06<br>2.65E-07<br>4.82E-06<br>5.47E-09<br>0.9764<br>0.7786<br>0.0219<br>0.7233<br>0.0086<br>0.0111
6B	Neurite Lenght	SFM, n = 4<br>FGF21, n = 4	WT vs. KO	8.29E-04

6C	Number of neurites per lenght intervals	SFM, n = 4<br>FGF21, n = 4	0-20: SFM vs. FGF21<br>20-40: SFM vs. FGF21<br>40-60: SFM vs. FGF21<br>60-80: SFM vs. FGF21<br>>80: SFM vs. FGF21	0.0518<br>7.51E-04<br>0.1933<br>4.65E-04<br>8.12E-04
6D	Klb expression in FACS-isolated GnRH neurons	NEG, n = 3<br>POS, n= 3	WT vs. KO	0.0265
6E	GnRH ELISA	WT, n = 5<br>KO, n = 5	WT: basal vs. 1<br>WT: basal vs. 10<br>WT: basal vs. 100<br>WT: basal vs. 1000<br>WT: 1 vs. 10<br>WT: 1 vs. 100<br>WT: 1 vs. 1000<br>WT: 10 vs. 100<br>WT: 10 vs. 1000<br>WT: 100 vs. 1000<br>KO: basal vs. 1<br>KO: basal vs. 10<br>KO: basal vs. 100<br>KO: basal vs. 1000<br>KO: 1 vs. 10<br>KO: 1 vs. 100<br>KO: 1 vs. 1000<br>KO: 10 vs. 100<br>KO: 10 vs. 1000<br>KO: 100 vs. 1000	0.0036<br>3.39E-06<br>3.65E-05<br>1.58E-05<br>0.2858<br>0.8002<br>0.6149<br>0.9981<br>1.0000<br>1.0000<br>0.9875<br>0.8989<br>0.9940<br>0.6826<br>1.0000<br>1.0000<br>0.9987<br>1.0000<br>1.0000<br>0.9966

EV1B	Western	n = 3<br>(for each conditions)	Expression: KLB R309W vs WT<br>Maturation: KLB R309W vs WT<br>Expression: KLB A547T vs WT<br>Maturation: KLB A547T vs WT<br>Expression: KLB R309Q vs WT<br>Maturation: KLB R309Q vs WT<br>Expression: KLB K815E vs WT<br>Maturation: KLB K815E vs WT<br>Expression: KLB F777delF vs WT<br>Maturation: KLB F777delF vs WT<br>Expression: KLB R424C vs WT<br>Maturation: KLB R424C vs WT<br>Expression: KLB L1011P vs WT<br>Maturation: KLB L1011P vs WT	9.89E-05<br>0.0470<br>0.0976<br>0.1436<br>0.9745<br>0.6910<br>0.1014<br>0.0066<br>0.8859<br>0.1249<br>0.3662<br>0.0017<br>0.8568<br>0.0122
EV2A	Vaginal opening on body weight	WT n = 11<br>KO n = 12	WT vs. KO	9.74E-05
EV2B	First estrus on body weight	WT n = 7<br>KO n = 5	WT vs. KO	0.0022
EV2C	Uterine weight	WT Di n = 6<br>WT Pro, n = 8<br>KO Di, n = 5<br>KO Pro, n = 5	Di: WT vs. KO<br>Pro: WT vs. KO	0.1286<br>0.6606
EV2D	Body lenght	WT n = 15<br>KO n = 10	WT vs. KO	2.72E-05
EV2E	Lean mass (% BW)	WT n = 23<br>KO n = 20	WT vs. KO	0.5026
EV2F	Daily food intake	WT n = 10<br>KO n = 10	WT vs. KO	0.8945

EV2G	Daily food intake (% BW)	WT n = 10<br>KO n = 10	WT vs. KO	0.8759
EV2J	GTT	WT n = 13<br>KO n = 11	0 : WT vs. KO<br>15 : WT vs. KO<br>30 : WT vs. KO<br>60 : WT vs. KO<br>90 : WT vs. KO<br>120 : WT vs. KO	0.3729<br>0.8303<br>0.9407<br>0.9599<br>0.9258<br>0.7804
EV2K	Insulin and Leptin plasma level	WT n = 5<br>KO n = 4	Insulin: WT vs. KO<br>Leptin: WT vs. KO	0.6278<br>0.7936
EV2L	Cholesterol plasma levels	WT n = 8<br>KO n = 3	Chol: WT vs. KO<br>HDL-Ch: WT vs. KO<br>LDL-Ch: WT vs. KO	0.2787<br>0.4531<br>0.1566
EV3C	Time per estrous phase	WT n = 16<br>HET n = 16	Diestrus: WT vs. HET<br>Pro: WT vs. HET<br>Estrus: WT vs. HET	6.74E-04<br>0.5771<br>0.0038
EV3D	Time per estrous phase	WT n = 17<br>KO n = 13	Diestrus: WT vs. KO<br>Pro: WT vs. KO<br>Estrus: WT vs. KO	6.12E-11<br>0.0250<br>1.31E-06
EV3E	LH on estrous cycle	WT Di n = 8<br>WT Es, n = 8<br>HET Di, n = 6<br>HET Es, n = 10	WT: Di vs. Es<br>HET: Di vs. Es	1.03E-04<br>0.6251
EV3F	LH on estrous cycle	WT Di n = 15<br>WT Es, n = 16<br>KO Di, n = 15<br>KO Es, n = 14	WT: Di vs. Es<br>KO: Di vs. Es	0.0216<br>0.9125
EV3G	Pregnancy rate	WT, n = 12<br>HET, n = 12	WT vs. KO	0.0405

EV4A	Gene expression MBH	WT, n = 5<br>HET, n = 5	Kiss1, WT vs. KO<br>Lepr, WT vs. KO<br>Npy, WT vs. KO<br>Pomc, WT vs. KO	0.3035<br>0.4206<br>0.2472<br>0.6482
EV4B	Gene expression POA	WT, n = 5<br>HET, n = 5	Gpr54, WT vs. KO<br>Kiss1, WT vs. KO<br>Lepr, WT vs. KO<br>Avp, WT vs. KO	0.2437<br>0.4222<br>0.2880<br>0.1145
EV4C	GnRH ELISA ME	WT, n = 4<br>HET, n = 4	WT vs. KO	0.2577

Appendix References:

Cole LW, Sidis Y, Zhang C, Quinton R, Plummer L, Pignatelli D, Hughes VA, Dwyer AA, Raivio T, Hayes FJ, Seminara SB, Huot C, Alos N, Speiser P, Takeshita A, Van Vliet G, Pearce S, Crowley WF, Jr., Zhou QY, Pitteloud N (2008) Mutations in prokineticin 2 and prokineticin receptor 2 genes in human gonadotrophin-releasing hormone deficiency: molecular genetics and clinical spectrum. The Journal of clinical endocrinology and metabolism 93**:** 3551-3559

de Roux N, Young J, Misrahi M, Genet R, Chanson P, Schaison G, Milgrom E (1997) A family with hypogonadotropic hypogonadism and mutations in the gonadotropin-releasing hormone receptor. The New England journal of medicine 337**:** 1597-1602

Falardeau J, Chung WC, Beenken A, Raivio T, Plummer L, Sidis Y, Jacobson-Dickman EE, Eliseenkova AV, Ma J, Dwyer A, Quinton R, Na S, Hall JE, Huot C, Alois N, Pearce SH, Cole LW, Hughes V, Mohammadi M, Tsai P et al (2008) Decreased FGF8 signaling causes deficiency of gonadotropin-releasing hormone in humans and mice. The Journal of clinical investigation 118**:** 2822-2831

Pitteloud N, Meysing A, Quinton R, Acierno JS, Jr., Dwyer AA, Plummer L, Fliers E, Boepple P, Hayes F, Seminara S, Hughes VA, Ma J, Bouloux P, Mohammadi M, Crowley WF, Jr. (2006) Mutations in fibroblast growth factor receptor 1 cause Kallmann syndrome with a wide spectrum of reproductive phenotypes. Molecular and cellular endocrinology 254-255**:** 60-69

Raivio T, Falardeau J, Dwyer A, Quinton R, Hayes FJ, Hughes VA, Cole LW, Pearce SH, Lee H, Boepple P, Crowley WF, Jr., Pitteloud N (2007) Reversal of idiopathic hypogonadotropic hypogonadism. The New England journal of medicine 357**:** 863-873

Santhakumar A, Balasubramanian R, Miller M, Quinton R (2014) Reversal of isolated hypogonadotropic hypogonadism: long-term integrity of hypothalamo-pituitary-testicular axis in two men is dependent on intermittent androgen exposure. Clinical endocrinology 81**:** 473-476
