# Supplementary material for: KLB, encoding β‐Klotho, is mutated in patients with congenital hypogonadotropic hypogonadism
Source: EMBO Mol Med. 2017 Jul 28;9(10):1379–97. doi: 10.15252/emmm.201607376 (PMC5623842; doi:10.15252/emmm.201607376)
Supplement: Supplementary file 2 — Expanded View Figures PDF [file EMMM-9-1379-s002.pdf]

Expanded View Figures

Figure EV1. Putative pathogenic variants in *KLB* identified in congenital hypogonadotropic hypogonadism.

A Identified KLB variants and conservation of affected KLB residues. Schematic of β-Klotho with identified mutations in CHH probands and amino acid conservation data on mouse, chicken, *Xenopus*, zebrafish, and human Klotho. GH1 denotes glycosyl hydrolase-1; TM denotes transmembrane domain. Gray dots indicate the known glycosylation sites.

B Expression and maturation of WT and KLB mutants. Cell lysates were subjected to PNGase (P) or EndoH (E) digestion and then processed for KLB immunoblotting using anti-HA antibodies. Overall expression levels were determined by PNGase-treated bands. Receptor maturation levels were calculated by the fraction of the EndoH-resistant band (mature) out of the total KLB immunoreactivity of EndoH-treated samples, and mutants were compared to WT using unpaired *t*-test. Blots are cropped for the purpose of presentation and indicated by black line. The experiments were repeated three times and data were plotted as mean ± SEM. U: untreated sample. *$P < 0.05$, **$P < 0.01$, ***$P < 0.001$.

 © 2017 The Authors

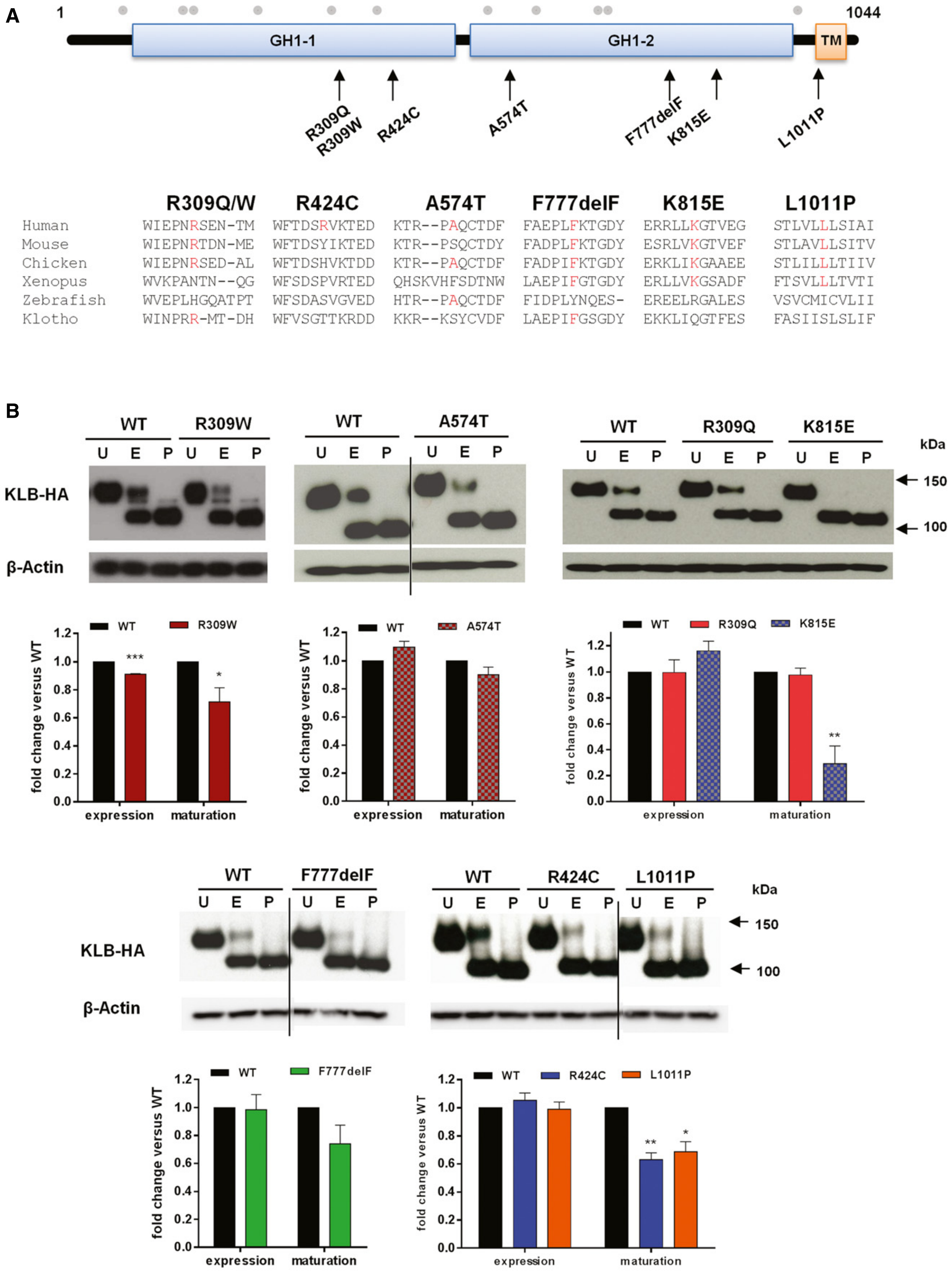
A
1
1044
GH1-1
GH1-2
TM
R309Q
R309W
R424C
A574T
F777delF
K815E
L1011P
R309Q/W
R424C
A574T
F777delF
K815E
L1011P
Human WIEPNRSEN-TM WFTDSRVKTED KTR--PAQCTDF FAEPLFKTGDY ERRLLKGTVEG STLVLLLSIAI
Mouse WIEPNRTDN-ME WFTDSYIKTED KTR--PSQCTDY FADPLFKTGDY ESRLVKGTVEF STLAVLLSITV
Chicken WIEPNRSED-AL WFTDSHVKTDD KTR--PAQCTDF FADPIFKTGDY ERKLIKGAAEE STLILLLTIIV
Xenopus WVKPANTN--QG WFSDSPVRTED QHSKVHFSDTNW LAEPIFGTGDY ERLLVKGSADF FTSVLLLTVTI
Zebrafish WVEPLHGQATPT WFSDASVGVED HTR--PAQCTDF FIDPLYNQES- EREELRGALES VSVCMICVLII
Klotho WINPRR-MT-DH WFVSGTTKRDD KKR--KSYCVDF LAEPIFGSGDY EKKLIQGTFES FASIISLSLIF
B
WT
R309W
A574T
R309Q
K815E
F777delF
R424C
L1011P
U E P
KLB-HA
β-Actin
kDa
150
100
fold change versus WT
expression
maturation
***
*
**
0.0
0.2
0.4
0.6
0.8
1.0
1.2

Figure EV1.

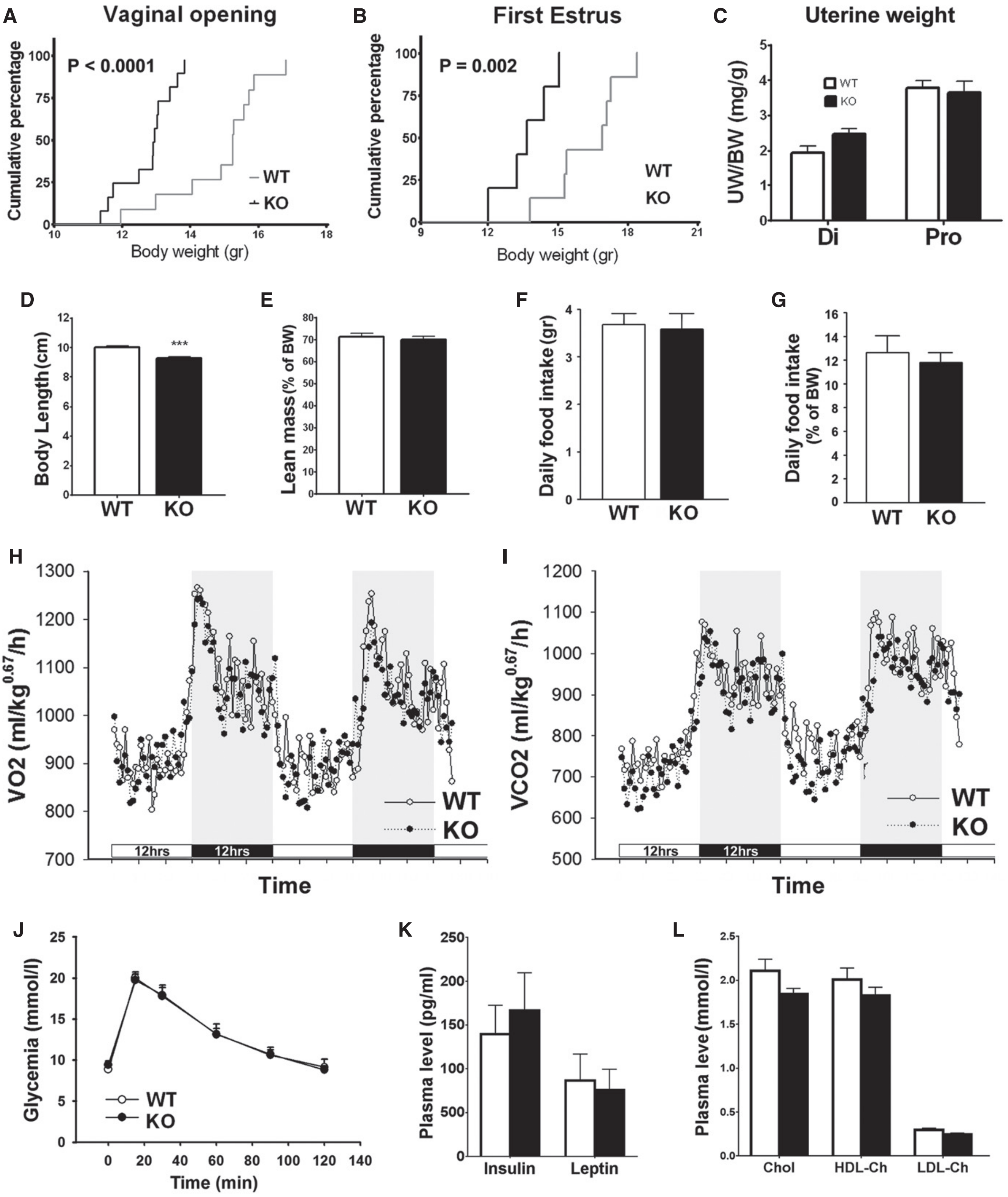

Figure EV2.

 © 2017 The Authors

Figure EV2. Extended reproductive and metabolic features in KlbKO mice.

A, B Cumulative percentage of vaginal opening (WT: n = 11; KO: n = 12) and first estrus (WT: n = 7; KO: n = 5) as a function of body weight, Gehan–Breslow–Wilcoxon test.

C Normalized uterine weight expressed as the uterine/body weight ratio (mg/g) in the diestrus (Di WT: n = 6; Di KO: n = 5) and proestrus (Pro WT: n = 8; Pro KO: n = 5) phases of the estrous cycle, unpaired t-test.

D Body length of adult wild-type KlbKO compared to littermates (WT: n = 15; KO: n = 10), unpaired t-test; ***$P < 0.001$.

E Lean mass expressed as a percentage of body weight of adult females (WT: n = 23; KO: n = 20), unpaired t-test.

F, G Raw and normalized (percentage of body weight) daily food intake (WT: n = 10; KO: n = 10), unpaired t-test.

H, I Energy expenditure evaluated using metabolic cages: measurement of oxygen consumption (VO_2) and carbon dioxide production (VCO_2) (WT: n = 10; KO: n = 10). Horizontal dark bars represent 12 h dark phase.

J Glycemia during a glucose tolerance test (WT: n = 13; KO: n = 11), unpaired t-test.

K, L Plasmatic levels of insulin, leptin (WT: n = 5; KO: n = 4), free, and bound cholesterol (WT: n = 8; KO: n = 3), unpaired t-test.

Data information: Values shown are mean ± SEM.

© 2017 The Authors

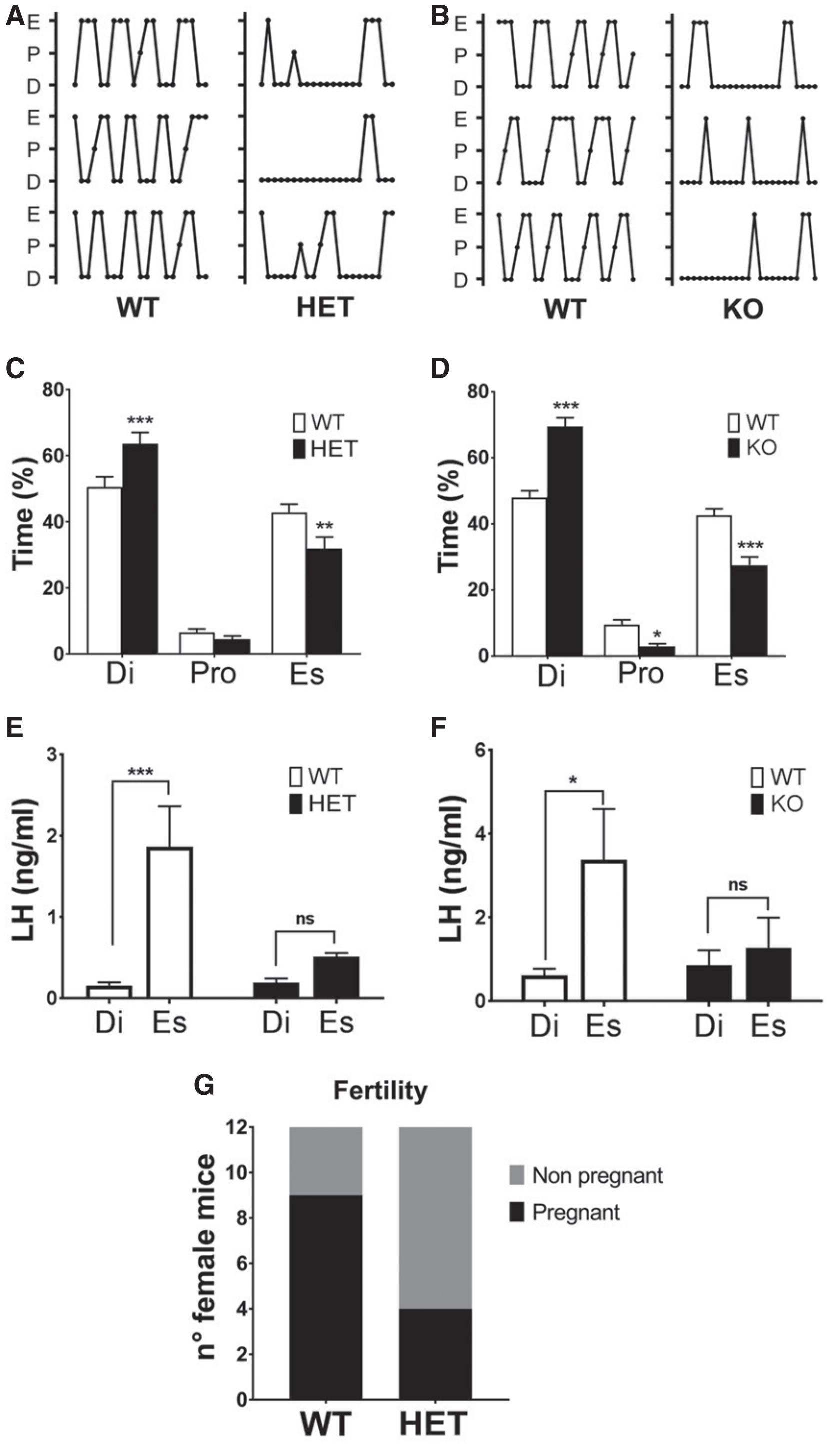

Figure EV3. KlbHET mice exhibit altered estrous cycle and subfertility.

A, B Comparison of representative estrous cycle patterns of 3- to 4-month-old WT vs. HET (A) and WT vs. KO (B) females, demonstrating marked alteration in both KlbHET and KlbKO.

C, D (C) Quantification of time spent in different estrous cycle phases in WT (n = 16) vs. KlbHET (n = 16) and (D) in WT (n = 17) vs. KlbKO (n = 13) adult females, unpaired t-test.

E, F Altered LH amplitude during the estrous cycle of KlbHET and KlbKO female mice. (E) Reduced LH levels during the estrous phase of KlbHET mice (HET Di: n = 6; HET Es: n = 10; WT Di: n = 8; WT Es: n = 8). Di: diestrus; Es: estrus; Pro: proestrus. (F) Reduces LH levels during the estrous phase of KlbKO mice (WT Di: n = 15; WT Es: n = 16; KO Di: n = 15; KO Es: n = 14). Data were analyzed by two-way ANOVA followed by Sidak's multiple comparisons test.

G Fertility evaluated as number of pregnant vs. non-pregnant in female KlbHET (n = 12) and WT littermates (n = 12) in a short-term mating protocol, chi-squared test.

Data information: Values shown are mean ± SEM; $*P < 0.05$, $**P < 0.01$, $***P < 0.001$.

 © 2017 The Authors

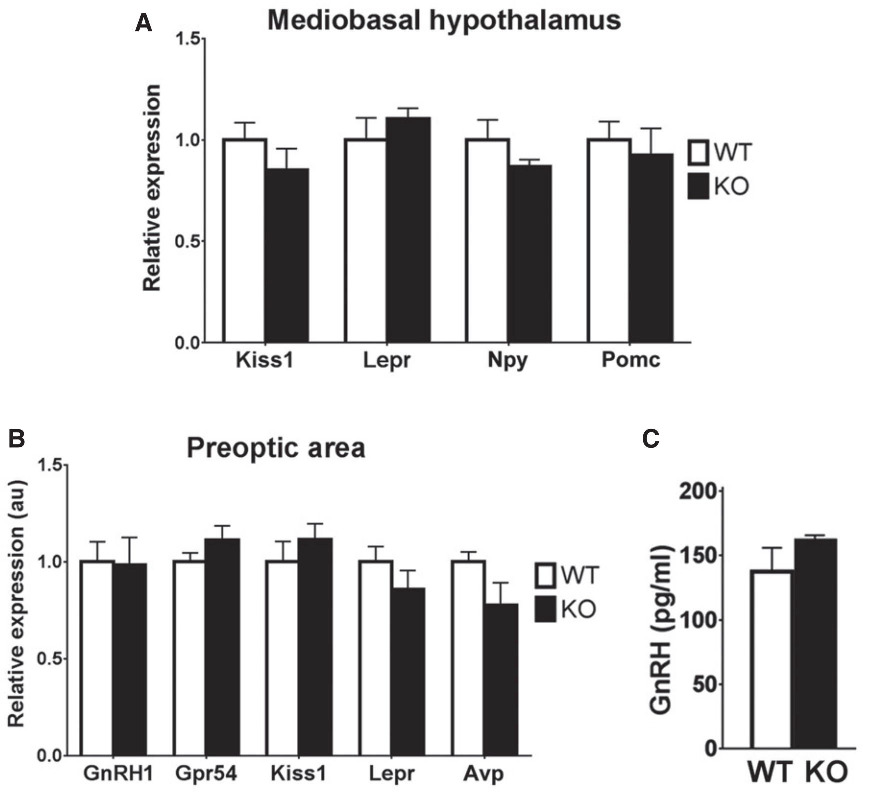

Figure EV4. Loss of *Klb* in female mice does not impair the expression of key hypothalamic genes including GnRH.

A, B Gene expression profiles on MBH and POA hypothalamic microdissection from KlbKO and WT adult brains (n = 5 per group). Differences between groups were assessed using unpaired t-test. Values are shown as mean ± SEM.

C Quantification of GnRH peptide content by ELISA in conditioned medium from median eminence explant cultures treated with 0.05 M KCl in order to induce the release of the entire GnRH vesicular pool (WT: n = 4; KO: n = 4). Values are shown as mean ± SEM.

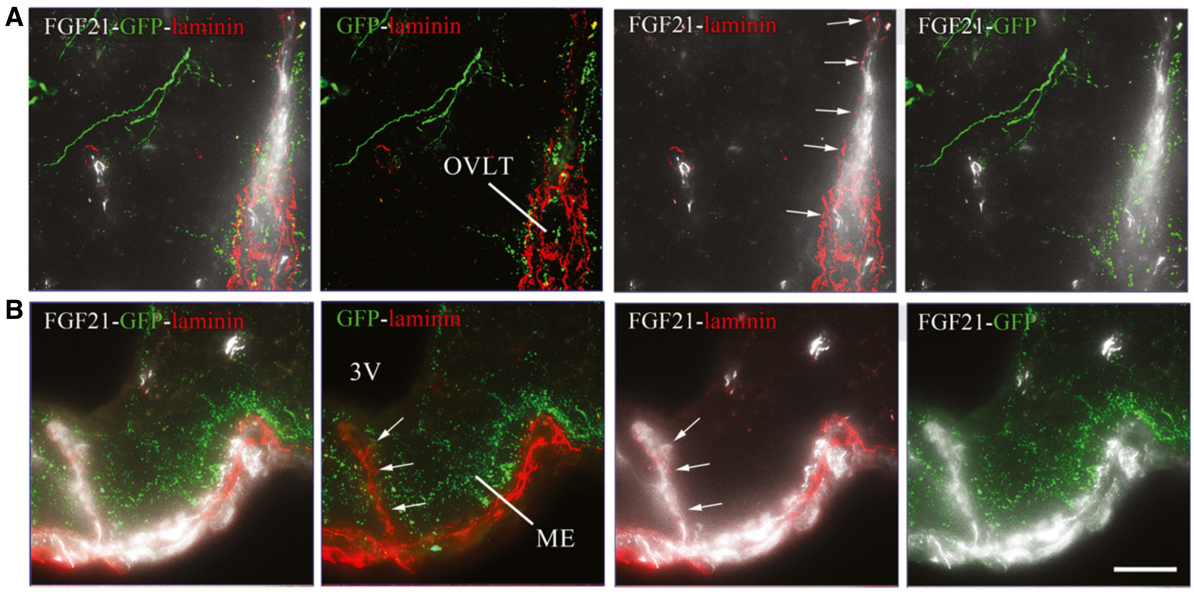

Figure EV5. FGF21 access to GnRH neurons by fenestrated vessels.

A Representative photomicrographs of the preoptic region showing GnRH neurons (GFP, green) and laminin immunoreactivity surrounding the blood vessels of the OVLT (arrows, red) labeled by fluorescent rFGF21 (5 nmol/animal, white staining). 3V, third ventricle; OVLT, organum vasculosum laminae terminalis.

B Representative photomicrographs showing laminin immunoreactivity surrounding the blood vessels of the ME (arrows, red) labeled by fluorescent rFGF21 (5 nmol/animal, white staining) in the tuberal region of the hypothalamus. Scale bar 40 µm (for A and B).

© 2017 The Authors
