# Supplementary figures and images for: KLB, encoding β‐Klotho, is mutated in patients with congenital hypogonadotropic hypogonadism
Source: EMBO Mol Med. 2017 Jul 28;9(10):1379–97. doi: 10.15252/emmm.201607376 (PMC5623842; doi:10.15252/emmm.201607376)

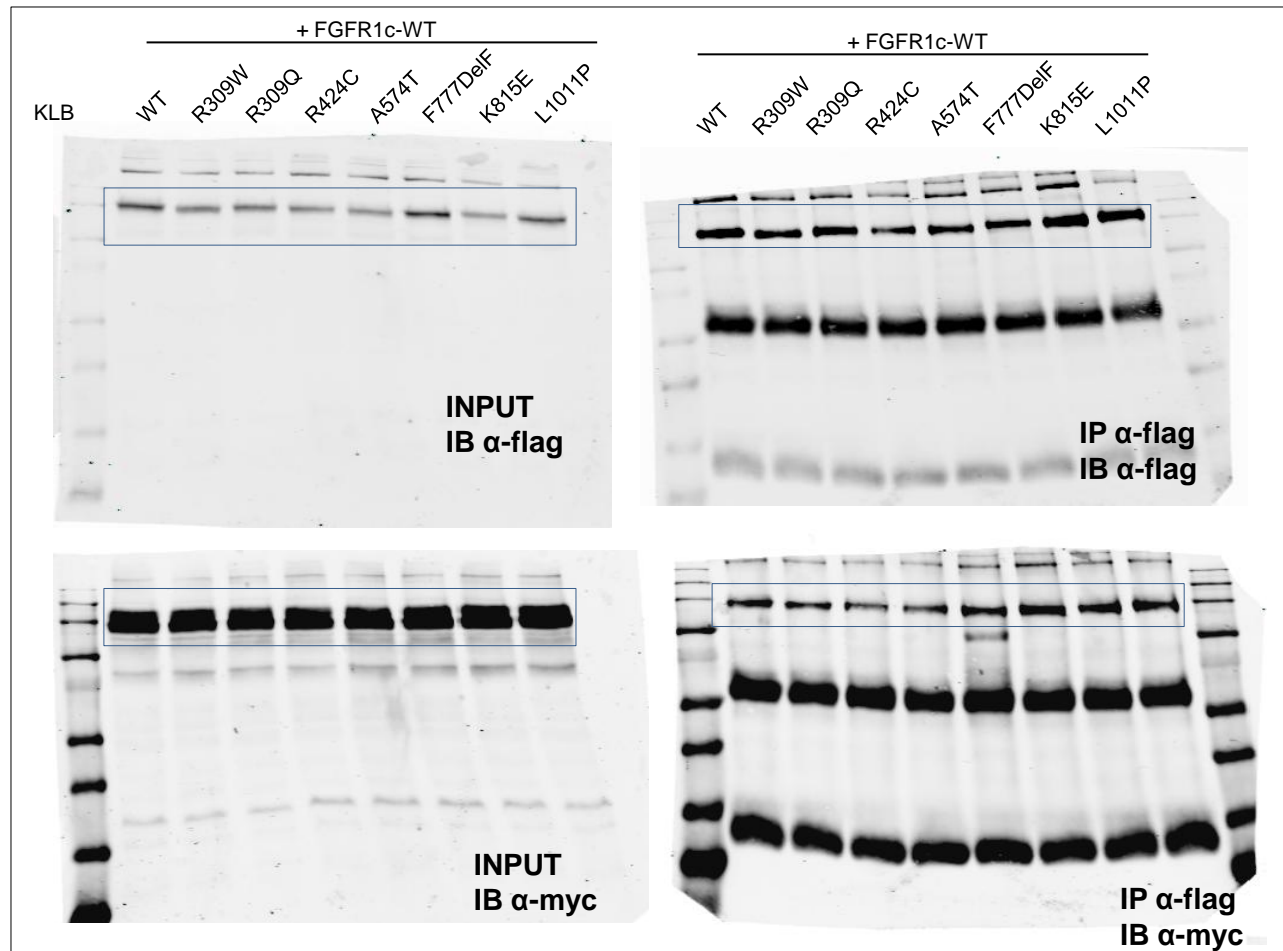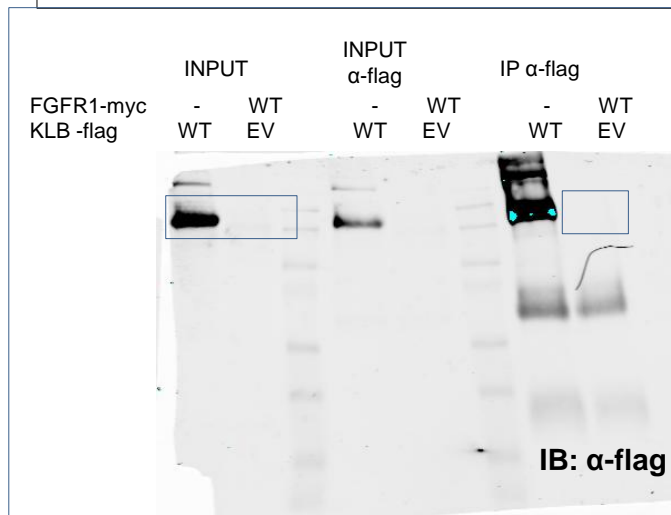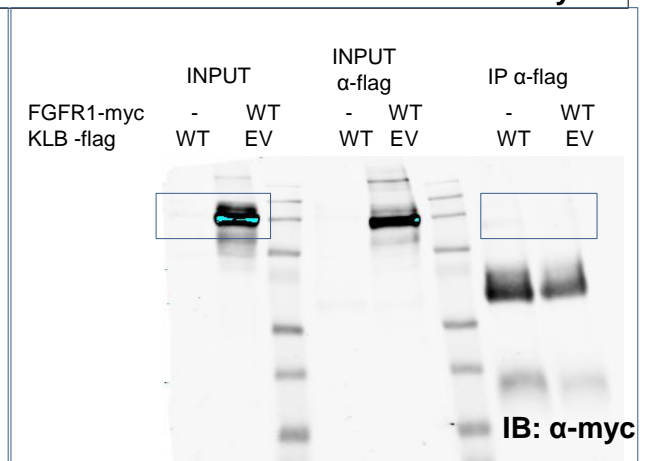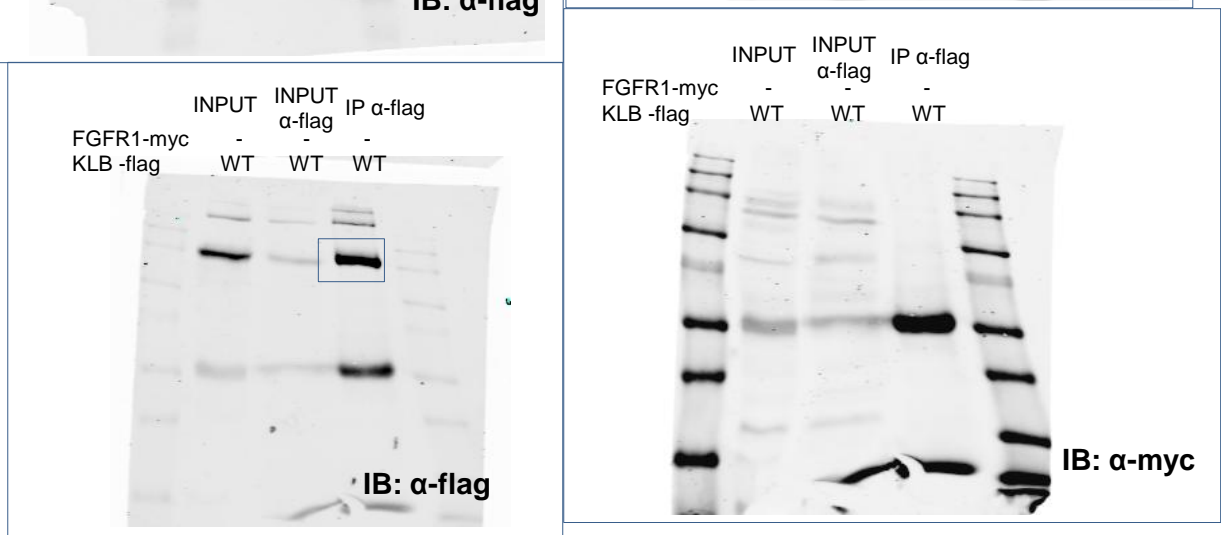

Odyssey system: saturated bands are shown in blue

Supplement: Supplementary file 4 — Source Data for Figure 2 [file EMMM-9-1379-s003.pdf]
